# Unveiling the drivers of patient satisfaction in the United States hospitals: Assessing quality indicators across regions

**DOI:** 10.1371/journal.pone.0324737

**Published:** 2025-06-11

**Authors:** Man Hung, Sharon Vu, Eric S. Hon, Logan Reese, Jacob Gardner, Martin S. Lipsky

**Affiliations:** 1 College of Dental Medicine, Roseman University of Health Sciences, South Jordan, Utah, United States of America; 2 The Wharton School, University of Pennsylvania, Philadelphia, Pennsylvania, United States of America; 3 Division of Public Health, University of Utah, Salt Lake City, Utah, United States of America; 4 Department of Orthopaedics, University of Utah, Salt Lake City, Utah, United States of America; 5 Department of Veterans Affairs Medical Center, Salt Lake City, Utah, United States of America; 6 School of Medicine, Boston University, Boston, Massachusetts, United States of America; 7 Lake Erie College of Osteopathic Medicine, Erie, Pennsylvania, United States of America; 8 Department of Economics, University of Chicago, Chicago, Illinois, United Staes of America; 9 Idaho College of Osteopathic Medicine, Meridian, Idaho, United States of America; 10 Institute on Aging, Portland State University, Portland, Oregon, United States of America; The University of Warwick, UNITED KINGDOM OF GREAT BRITAIN AND NORTHERN IRELAND

## Abstract

**Introduction:**

Patient satisfaction in the UnitedStates (U.S.) healthcare varies regionally due to cultural, socioeconomic, and infrastructure differences. The Hospital Consumer Assessment of Healthcare Providers and Systems (HCAHPS) survey measures patient satisfaction across several aspects, including staff responsiveness and hospital environment. This survey influences Medicare reimbursements and helps ensure equitable, high-quality care nationwide. Analyzing these results is crucial for enhancing patient-centered care and understanding regional disparities.

**Methods:**

This study analyzed HCAHPS data from 3,286 U.S. hospitals from July 1, 2021, to June 30, 2022. Hospitals were stratified by region. The categories analyzed included cleanliness, communication, staff responsiveness, medication information, discharge processes, care transition, overall rating, quietness, and hospital recommendations. Kruskal Wallis tests and heat maps were used to compare and visualize regional differences.

**Results:**

The analysis revealed significant regional differences in hospital performance across the U.S. (p < 0.05). The Midwest consistently scored the highest in hospital performance metrics, while the “Other” region reported the lowest scores with discharge information, 12.91 percentage points (pp) lower than the Midwest. The communication about medicines indicator scored the lowest across all regions, with the Midwest the best at 76.88, 1.67 pp higher than the West and 9.94 pp higher than the “Other” region. State-level heatmaps highlighted disparities, with New York and South Carolina performing poorly, while South Dakota earned 5-star ratings for overall hospital ratings.

**Conclusions:**

U.S. healthcare service ratings demonstrate regional disparities, with the Midwest scoring highest overall. The study identified specific areas needing improvement in lower-performing states, contrasting with strong performances in others. These findings can guide policymakers in enhancing national healthcare quality by addressing regional challenges and learning from high-performing states. Understanding these disparities is crucial for improving patient-centered care, reducing quality gaps, and ensuring equitable access to high-quality healthcare across the U.S.

## Introduction

Measuring patient experience and satisfaction are pivotal for enhancing healthcare services [1]. Research on these indicators across the United States (U.S.) is essential because of regional differences in cultural attitudes, socioeconomic status, and healthcare infrastructure that can affect patient expectations and perceptions of care. Studying regional differences can help researchers to identify unique regional needs, which are crucial for tailoring healthcare services. Evidence suggests that patient satisfaction is linked to clinical outcomes, patient retention, and medical malpractice claims [2]. By developing benchmarks and comparing patient satisfaction scores, hospitals and healthcare systems can identify best practices and learn from areas with higher patient satisfaction [3]. Data sharing also fosters a culture of continuous improvement and shared learning within the healthcare community, which can improve patient care and healthcare efficiency.

Studying regional differences in patient satisfaction also informs healthcare policy and resource allocation. Lower satisfaction scores in certain regions could signal the need for regional policy changes or additional resources, including more funding for hospital improvements or staff training programs [4]. Variations in satisfaction might reflect unequal healthcare access or differences in quality of care among populations and stimulate healthcare providers and policymakers to develop targeted strategies to promote health equity [5]. By analyzing patient satisfaction across regions, healthcare providers also gain insight into aligning their services with patient needs and preferences and improving the overall quality of care [6].

Patient satisfaction can be measured using several methodologies, each offering distinct strengths and insights into various aspects of the healthcare experience. Successfully capturing and enhancing patient experiences requires metrics that accurately mirror the concerns and priorities of patients. Quantitative survey methods are commonly used in patient satisfaction research since surveys can handle large sample sizes, enhancing the generalizability of the findings [7]. This approach allows for robust statistical analysis, facilitating the exploration of patterns, relationships, and trends using patient satisfaction data.

The Hospital Consumer Assessment of Healthcare Providers and Systems (HCAHPS) survey is an example of a quantitative tool for patient satisfaction. HCAHPS is the first national, standardized, publicly reported survey of patients’ perspectives of hospital care. First administered by CMS in 2006 and publicly reported since 2008, HCAHPS provides an opportunity to compare hospitals locally, regionally, and nationally on topics important to patients and consumers [8].

Participation in the HCAHPS survey differs among hospitals. The Inpatient Prospective Payment System (IPPS) mandates that acute care hospitals collect and submit HCAHPS data to qualify for a full annual payment update. In contrast, acute care hospitals not under the IPPS can choose to participate voluntarily. The IPPS, established under Section 1886(d) of the Social Security Act for Medicare Part A in the U.S. [9], pays hospitals based on a standardized rate per case or diagnosis, rather than the actual costs incurred during a patient’s stay [10]. Subsequent Medicare reimbursements are adjusted based on clinical processes, patient outcomes, and patient experiences. Notably, 2% of total Medicare reimbursements are influenced by HCAHPS survey results, meaning hospitals with lower HCAHPS scores receive reduced reimbursements from the Centers for Medicare & Medicaid Services (CMS) [11]. The Deficit Reduction Act of 2005 encouraged acute care hospitals to participate in the HCAHPS survey. Later, the Patient Protection and Affordable Care Act of 2010 further emphasized the importance of participation, especially for IPPS hospitals, in enhancing patient experience [12,13].

Analyzing patient satisfaction across different regions in the U.S. is crucial due to the country’s vast geographic and demographic diversity. States have unique cultural, economic, and healthcare infrastructure characteristics that influence patient expectations and perceptions of care [14–18]. For instance, socioeconomic factors can affect patients’ experiences and satisfaction with healthcare [19]. By stratifying patient satisfaction data across regions, researchers can uncover specific regional needs and preferences, enabling healthcare providers to tailor their services more effectively.

The HCAHPS survey results are publicly available and provide valuable insights into patient experience and satisfaction across different hospitals in the U.S. The current study aimed to analyze the determinants of patient satisfaction in hospitals across various regions in the U.S., utilizing HCAHPS survey data. This analysis should identify areas with lower satisfaction scores, helping public health policymakers and others to plan resource allocation and interventions to address deficiencies and improve overall healthcare quality.

## Methods

### Data

This study used data from two public datasets on data.cms.gov from July 1, 2021, to June 30, 2022: HCAHPS dataset and Prospective Payment System -Exempt Cancer Hospital Quality Reporting (PCHQR) Program dataset.

The HCAHPS dataset, compiled by CMS, includes patient satisfaction data from 3,275 hospitals. HCAHPS is administered to a random sample of adult inpatients between 48 hours and six weeks after discharge from medical, surgical, cancer, and maternity service areas. This survey is specifically tailored to gauge the inpatient hospital experience, covering dimensions of hospital care such as responsiveness of hospital staff, quality of discharge information, and a patient’s willingness to recommend the hospital [20]. It consists of 29 questions designed to gather feedback about a recent hospital stay. Nineteen questions address key aspects of the hospital experience, such as communication with health providers, responsiveness of hospital staff, cleanliness and quietness of the hospital environment, and the patient’s overall hospital rating [21]. Additionally, there are 7 demographic questions used for standardizing hospitals for analytical comparison and 3 remaining questions which are screening items that guide patients to subsequent relevant questions [22].

Each of the 19 HCAHPS patient satisfaction measures has scored answer choices. For example, the questions with answer choices, such as “Never,” “Sometimes,” “Usually,” and “Always,” are assigned scores of 1, 2, 3, and 4, respectively. CMS adjusts for survey mode and patient mix to adjust the raw scores using an equation to convert them into linear mean scores that range from 0 to 100 [23]. This leads to a 1,2,3, 4 or 5-star rating (1 = worst, 5 = best), where whole stars are assigned for each HCAHPS measure based on cut points derived from applying a clustering algorithm to the individual linear mean measure scores. Hospitals with at least 100 completed surveys over four quarters receive ratings. The PCHQR dataset, also compiled by CMS, includes the 19 HCAHPS patient satisfaction measures for Prospective Payment System Exempt Cancer Hospitals [24]. To participate, a hospital must be classified as a cancer hospital and exempt from the prospective payment system from its first cost reporting period on or after October 1, 1989. Eleven hospitals met these criteria [24]. Combined, these datasets provided adult inpatient data from 3,286 hospitals.

### Analytic approach

The analysis combined the two datasets using key facility details such as name, address, city, state, zip code, and county, along with HCAHPS measures and ratings, including HCAHPS Star Rating and HCAHPS linear mean score. Data on the number of completed surveys and survey response rate were also integrated. Rows lacking either the Patient Survey Star Rating or the HCAHPS linear mean score were excluded from the analysis. This filtration ensured that only data with both the linear mean score and star rating were retained for the following HCAHPS measures: Cleanliness, Nurse Communication, Doctor Communication, Staff Responsiveness, Communication About Medicines, Discharge Information, Care Transition, Overall Hospital Rating, Quietness, and Recommend Hospital. More details regarding HCAHPS can be found at http://www.hcahpsonline.org.

To enhance understanding of the quality indicators across geographic landscapes, states were categorized into regions: “Northeast,” “Midwest,” “South,” “West,” and “Other,” based on classifications from Wikipedia’s List of regions of the United States [25]. Table 1 categorizes states into these regional classifications.

**Table 1:** Regional Classification.

Region Classification	State	Number of Hospitals
Northeast	Connecticut, Maine, Massachusetts, New Hampshire, Rhode Island, Vermont, New Jersey, New York, Pennsylvania	492
Midwest	Illinois, Indiana, Michigan, Ohio, Wisconsin, Iowa, Kansas, Minnesota, Missouri, Nebraska, North Dakota, South Dakota	879
South	Delaware, Florida, Georgia, Maryland, North Carolina, South Carolina, Virginia, West Virginia, Alabama, Kentucky, Mississippi, Tennessee, Arkansas, Louisiana, Oklahoma, Texas	1,210
West	Arizona, Colorado, Idaho, Montana, Nevada, New Mexico, Utah, Wyoming, Alaska, California, Hawaii, Oregon, Washington	687
Other	District of Columbia, Puerto Rico, Virgin Islands, American Samoa, Guam, Northern Mariana Islands	18

Descriptive statistics were calculated for continuous variables, providing mean, standard deviation, minimum, and maximum values. For categorical variables, frequency counts (n) and percentages were calculated. A Kruskal-Wallis test was conducted to compare each hospital indicator’s linear mean scores, stratified by region. A *p*-value less than 0.05 was considered significant. Heat maps were created to represent patient satisfaction visually across the U.S. using star ratings. Heatmaps were generated in RStudio using the ggplot2, sf, ggspatial, maps, mapproj, gridExtra, and scales packages to visualize the distribution of star ratings for each hospital indicator across the U.S. Each state is color-coded based on the most common star rating for a specific hospital determinant among hospitals within that state. For instance, if most hospitals in a state have a 5-star rating, the state is shaded dark green, whereas a predominance of 1-star ratings results in a red fill. RStudio version 2023.06.1 + 524 was used for all statistical analyses.

## Results

The South had the largest number of reporting hospitals [1],(210), followed by the Midwest (879), the West (687), and the Northeast (492), with the “Other” category having the least [18] (see Table 1).

Table 2 summarizes the overall linear mean scores of all the states. Both doctor and nurse communications scored over 90 percentage points (pp). Communication about medicines scored the lowest at 75.34 pp.

**Table 2:** National patient satisfaction linear scores for HCAHPS.

Categories	Minimum	Maximum	Median	Mean	Std. Dev.
Cleanliness	67.00	99.00	86.00	85.26	4.54
Nurse communication	75.00	100.00	91.00	90.37	2.84
Doctor communication	74.00	100.00	90.00	90.21	2.81
Staff responsiveness	59.00	100.00	83.00	82.59	5.08
Communication about medicines	53.00	100.00	75.00	75.34	4.90
Discharge information	60.00	100.00	86.00	85.56	4.13
Care transition	65.00	91.00	80.00	80.24	3.26
Overall hospital rating	67.00	97.00	87.00	87.00	3.92
Quietness	60.00	98.00	82.00	81.62	5.45
Recommend hospital	57.00	98.00	87.00	86.17	5.08

Table 3 presents the linear mean scores for hospital indicators by U.S. regions. All hospital indicators showed significant regional differences (p < 0.05). The Midwest region displayed the highest average linear mean score for every hospital determinant. Conversely, except for quietness, the “Other” group scored the lowest among the regions for all other measures. The differences for “Other” compared to the highest scoring (Midwest) region ranged from 12.91 pp for discharge information to 2.57 pp for doctor communication. Communication about medicines scored the lowest in all regions, with the Midwest scoring 1.67 pp more than the next closest region (West), and with a difference of 9.94 pp more than the “Other” region.

**Table 3:** Linear mean score (standard deviation) of patient satisfaction indicators by regions in the United States.

Categories	Midwest	Northeast	South	West	Other	*p*-value
Cleanliness	86.27 (4.75)	84.96 (4.18)	84.54 (4.66)	85.53 (3.99)	82.89 (4.93)	1.33e-18
Nurse communication	91.47 (2.49)	90.32 (2.91)	90.04 (2.68)	89.69 (2.94)	85.39 (3.96)	1.27e-52
Doctor communication	90.96 (2.57)	89.88 (2.36)	90.26 (2.77)	89.45 (3.18)	88.39 (3.07)	4.84e-22
Staff responsiveness	84.48 (4.84)	81.44 (5.30)	81.93 (4.89)	82.34 (4.78)	75.61 (5.42)	1.56e-45
Communication about medicines	76.88 (4.77)	74.43 (4.47)	74.78 (4.91)	75.21 (4.73)	66.94 (6.05)	1.46e-33
Discharge information	87.30 (3.57)	85.65 (4.05)	84.58 (3.90)	85.27 (4.04)	74.39 (8.31)	5.38e-66
Care transition	81.45 (2.98)	79.72 (3.02)	80.03 (3.19)	79.55 (3.42)	76.56 (4.10)	9.48e-43
Overall hospital rating	88.18 (3.63)	86.07 (3.95)	86.70 (3.82)	86.84 (3.97)	81.22 (4.98)	2.33e-34
Quietness	83.29 (4.71)	78.02 (4.78)	83.40 (4.64)	78.97 (5.80)	79.83 (4.69)	3.96e-128
Recommend hospital	87.41 (4.74)	85.34 (5.13)	85.72 (4.94)	86.11 (5.34)	80.44 (6.20)	1.13e-22

**Fig 1:**
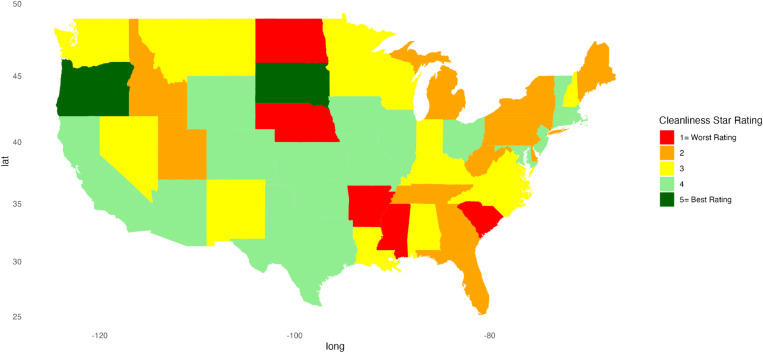
Heatmap of Cleanliness Star Rating Across States.

**Fig 2:**
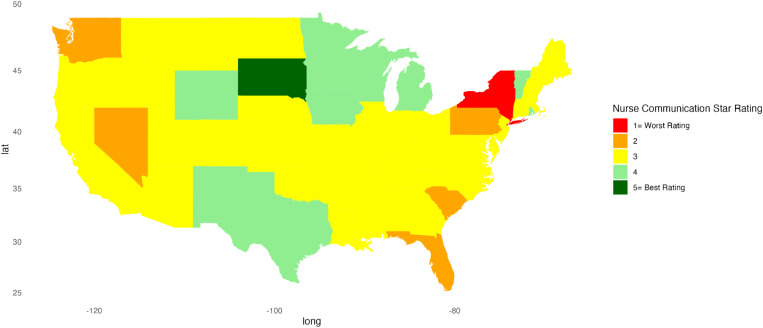
Heatmap of Nurse Communication Star Rating Across States.

**Fig 3:**
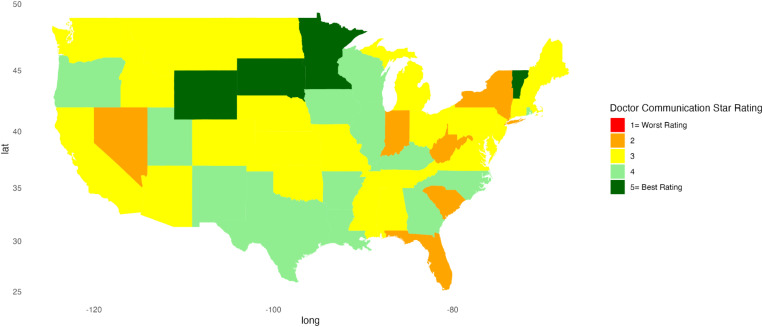
Heatmap of Doctor Communication Star Rating Across States.

**Fig 4:**
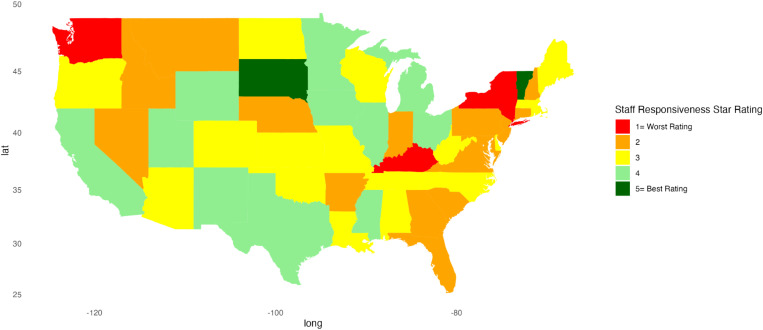
Heatmap of Staff Responsiveness Star Rating Across States.

**Fig 5:**
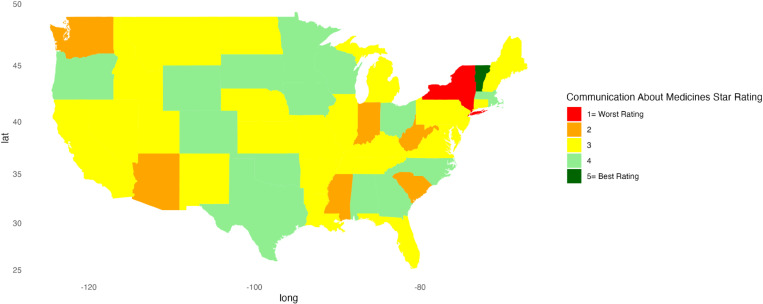
Heatmap of Communication About Medicines Star Rating Across States.

**Fig 6:**
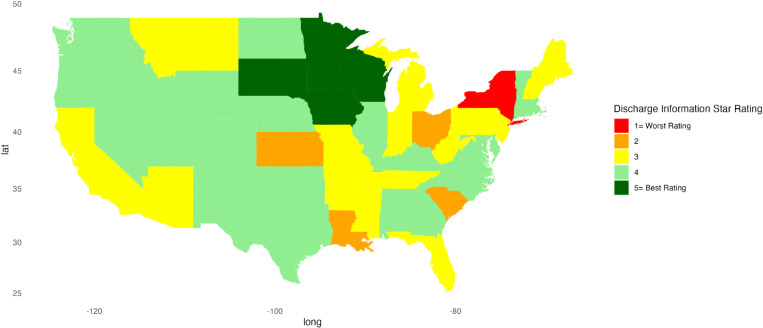
Heatmap of Discharge Information Star Rating Across States.

**Fig 7:**
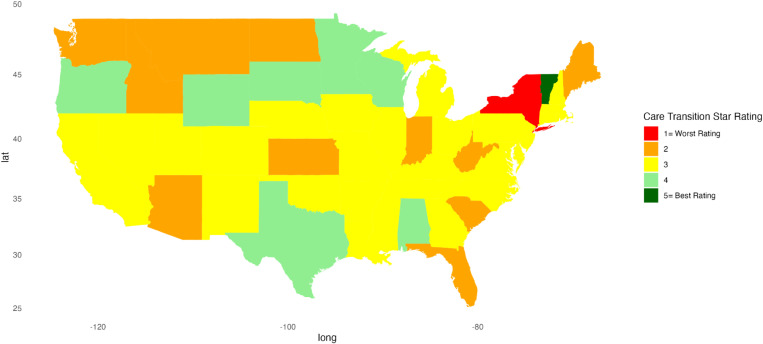
Heatmap of Care Transition Star Rating Across States.

**Fig 8:**
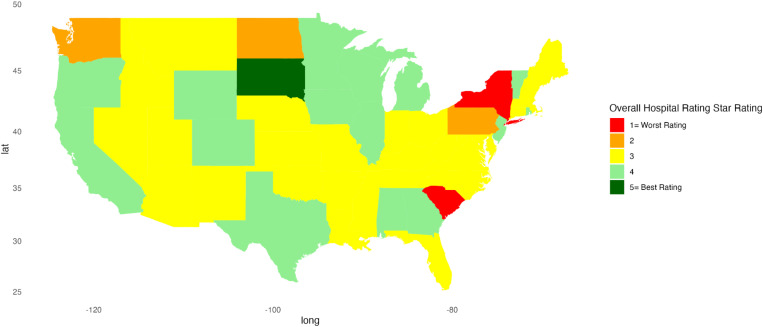
Heatmap of Overall Hospital Rating Star Rating Across States.

**Fig 9:**
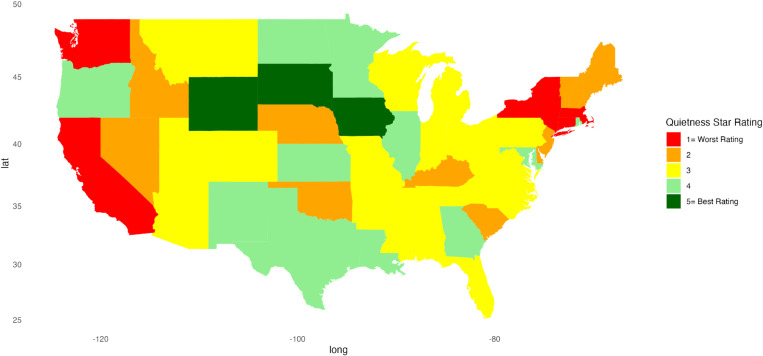
Heatmap of Quietness Star Rating Across States.

**Fig 10:**
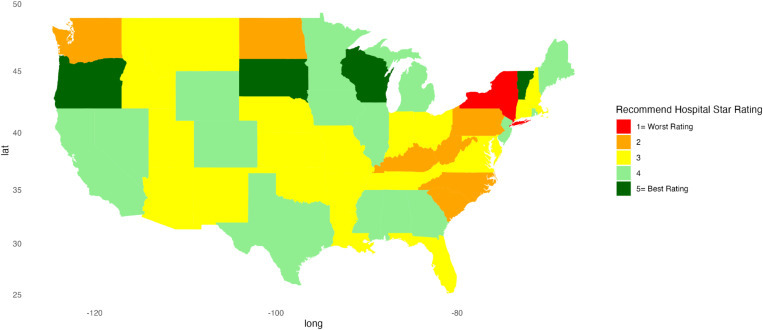
Heatmap of Recommend Hospital Star Rating Across States.

Figs 1–10 illustrate state-by-state heatmaps summarizing healthcare service ratings. Each state was colored according to the most common star rating for a specific hospital determinant. For example, a state with more hospitals receiving a 5-star rating was filled with dark green, while a state with more hospitals receiving a 1-star rating was filled with red. Categories include cleanliness, nurse and doctor communication, staff responsiveness, medication communication, discharge information, care transition, overall hospital rating, quietness, and hospital recommendations. The heatmaps contrast poorly performing states like New York and South Carolina with high achievers such as South Dakota, which earned 5-star ratings for their overall hospital ratings. Of note, only Vermont earned a 5-star rating for communication about medicines (Fig 5) and care transition (Fig 7). Table 4 presents the lowest and highest performing states across various categories of patient experience.

**Table 4:** Lowest and highest performing states across various categories of patient experience.

Categories	Lowest (1-star, or 2-star) performing states	Highest (5-star) performing states
Cleanliness	Arkansas, Mississippi, Nebraska, North Dakota, South Carolina	Oregon, South Dakota
Nurse Communication	New York	South Dakota
Doctor Communication	Florida, Indiana, Nevada, New York, South Carolina, West Virginia	Minnesota, South Dakota, Vermont, Wyoming
Staff Responsiveness	Kentucky, New York, Washington	South Dakota, Vermont
Communication about Medicines	New York	Vermont
Discharge Information	New York	Iowa, Minnesota, South Dakota, Wisconsin
Care Transition	New York	Vermont
Overall Hospital Rating	New York, South Carolina	South Dakota
Quietness	California, Connecticut, Massachusetts, New York, Oregon	Iowa, South Dakota, Wyoming
Recommending a Hospital	New York	Oregon, South Dakota, Vermont, Wisconsin

## Discussion

The standardized national HCAHPS tool enables meaningful hospital and regional comparisons. This study found significant regional variations in all measured hospital indicators (p < 0.05). The Midwest performed best, recording the highest average linear mean scores for nearly all hospital determinants, including cleanliness (86.27), nurse communication (91.47), and overall hospital rating (88.18). For the quietness indicator the South scored the best (83.40). In contrast, the “Other” region scored the lowest, with significant gaps in staff responsiveness (75.61), communication about medicines (66.94), and discharge information (74.39). Another national study also found that the Midwest scored higher than other regions for inpatient communication about medicines [26]. However, Mullings and Sankaranarayanan studied 171 hospitals and found no significant differences in patient satisfaction across geographic regions [27]. Differences in healthcare infrastructure, cultural values, patient expectations, economic conditions, and state policies are possible reasons why the Midwest scored better. Surprisingly, few high-quality studies examine interventions to improve patient satisfaction [28].

The triple aim framework provides a foundation for improving healthcare quality. A core component of the triple value aim is patient satisfaction [29,30]. While the goal that individuals should be satisfied with their care alone might justify its inclusion, positive associations of patient satisfaction with clinical effectiveness support the case for including it as a quality measure of healthcare [31–36]. A reassuring finding was that for overall satisfaction and willingness to recommend the hospital, the four major regions of the U.S. demonstrated moderately high levels of satisfaction. For these two key summation categories, only a difference of about 2 pp separated the highest and lowest regional scores. Even so, our findings suggest room for improvement in several categories and states.

Communication about medications and discharge information scored the lowest for all regions, highlighting the need for strategies to improve these parameters. Hospital discharge represents a vulnerable time for patients, and about 1 in 5 experience an adverse event after discharge or readmission within 30 days [37–39]. Discharge and medication communication likely work better when hospitals employ strategies to enhance healthcare professionals’ communication skills [40], empower patients [41], and adopt discharge and communication practices that impact patient satisfaction [40,42]. For example, incorporating a pharmacist and a discharge coordinator into medication communication and discharge planning can increase satisfaction scores and reduce readmissions [43–45]. Vermont’s leading performance suggests that research about why its hospitals performed better might inform a state like New York, which lagged in providing clear information about medications. Similarly, there was variation among states in the perception of discharge information, with New York rating worse than Iowa, Minnesota, South Dakota, and Wisconsin. Unraveling the factors and barriers that compromise medication and discharge instruction communication can impact patient outcomes, making efforts to enhance sharing information and improving low scores important [46–48].

Paradoxically, nurse and doctor communication earned high marks across regions, and while overall it scored better than other categories, states displayed notable disparities. New York was rated the lowest in nurse communication, highlighting a potential gap in provider-patient interactions. In contrast, South Dakota did well, suggesting this state might have better-trained, more attentive staff, or a culture that reinforced the value of effective communication. Similarly, New York’s lower rating for doctor communication contrasted sharply with the higher ratings of South Dakota and Vermont. The discrepancy between the ratings for communication with health professionals and information about medication and discharge planning is concerning but suggests opportunities for improving patient outcomes. Discharge planning and medication information are key elements of care transitions, and suboptimal transitions of care increase the risk of readmission and adverse drug events after discharge [49]. Others also found challenges and issues with medication reconciliation and discharge planning that might affect outcomes [45,50,51]. While our findings do not identify the reasons for differences, the variations among states and the positive ratings for doctor and nurse communication suggest there are opportunities to enhance care transitions that can benefit patients.

Another key finding was that the “Other” region (see Table 1) scored the lowest for all but one category, with the widest gaps in staff responsiveness, communication about medicines, and discharge information. This suggests a need to develop strategies to narrow these differences in performance and to consider directing more resources to the hospitals represented by this category.

The heat maps provide a comprehensive state-by-state analysis of healthcare service ratings across the U.S. and highlight significant disparities in healthcare quality. They reveal stark contrasts between low- and high-performing states for cleanliness, communication, staff responsiveness, and overall hospital ratings. One surprising finding was the variation in cleanliness standards since a clean hospital is often an unquestioned assumption. Arkansas, Mississippi, Nebraska, North Dakota, and South Carolina rated lowest, indicating significant opportunities for improvement, while states like Oregon and South Dakota rated highest, suggesting more effective management and protocols in maintaining hospital hygiene. A recent study found a 4.9 pp decrease in patient satisfaction with cleanliness during COVID-19 and suggests a heightened need to focus more on this category [52]. While pandemic-related staffing issues could account for lower scores, it may be that the COVID-19 pandemic increased awareness of hygiene and safety practices, such as handwashing and sanitization, making patients more critical of hospital cleanliness [53]. However, the importance of a clean environment cannot be overstated since cleaning reduces the incidence of healthcare-associated infections [54,55].

Hospitals across all regions scored the poorest in the staff responsiveness and care transition categories. Again, disparities among states suggest lower-performing states, like New York and South Carolina, might explore how they differ from better-performing states, such as South Dakota and Vermont.

Data on quietness and hospital recommendations provide insights into patient comfort and satisfaction. Overall, this was one of the lower-scoring categories, with a gap between high- and low-scoring regions (5.35 pp). Our findings are consistent with a recent review that identified few interventions that addressed quietness and suggested a need to develop and test processes to improve the hospital environment [43]. Drilling down to the state level, the lower ratings of California, Connecticut, Massachusetts, New York, and Oregon in quietness contrast with the higher ratings of Iowa, South Dakota, and Wyoming, suggesting environmental and operational differences.

### Limitations

This study was cross-sectional, which limits its applicability to future scenarios. While cross-sectional studies provide a snapshot in time, they do not account for the dynamic and evolving nature of patient satisfaction and healthcare standards. The findings may have been influenced by nonresponse bias. However, research on HCAHPS surveys reveals minimal evidence of nonresponse bias after accounting for patient case-mix adjustments [56].

Several factors, such as societal trends, technological advancements, policy changes, evolving patient expectations, and healthcare practices, influence patient satisfaction [57]. This study collected data over a relatively short timeframe and might not capture ongoing changes. New treatments and technologies can alter patient experiences and satisfaction levels [58]. Also, while regional differences probably reflect caregiving and quality that affect the patient experience, this study did not examine potential confounding variables such as cultural differences and hospital type that might influence survey responders. Finally, while the study’s findings are relevant for 2021–2022, they may not accurately predict future trends or account for evolving regional disparities.

Despite these limitations, this study remains informative. It provides valuable insight into the state of patient satisfaction across different regions in the U.S. during the specified period. The findings can serve as a benchmark for healthcare providers and policymakers, highlighting areas of excellence and those needing improvement.

### Implications

This study demonstrated disparities in patients’ experiences across hospital-referral regions, highlighting the need for a strategic and unified response to close the gaps in healthcare quality across the U.S. This might require adjusting resource allocation and targeting policy interventions to elevate underperforming regions. A coordinated approach, which aligns local healthcare policies, hospital management strategies, infrastructure investments, and staff training with national standards, might mitigate existing disparities in patient outcomes and satisfaction.

The data on staff responsiveness, medication communication, and care transition, including hospital discharge, underscore the pressing need to address issues such as understaffing and inadequate training across all regions. Prioritizing these areas will significantly enhance overall patient experiences and outcomes. Despite the inherent challenges, addressing these disparities is not just crucial—it is imperative for the future of national healthcare.

## Conclusions

Patient experience varied across regions, with the Midwest emerging as a top performer, demonstrating robust overall healthcare quality. Our study found moderately high satisfaction for overall hospital experience (88.18) and willingness to recommend the hospital (87.41) across regions, while communication about medications (76.88) and discharge information (87.30) scored the lowest. Disparities in staff responsiveness, medication communication, discharge information, and overall ratings exist across regions and states, suggesting the need for targeted interventions to bridge these gaps and improve patient satisfaction disparities across the U.S.
